# Functional and Structural Insights into Human PPARα/δ/γ Subtype Selectivity of Bezafibrate, Fenofibric Acid, and Pemafibrate

**DOI:** 10.3390/ijms23094726

**Published:** 2022-04-25

**Authors:** Akihiro Honda, Shotaro Kamata, Makoto Akahane, Yui Machida, Kie Uchii, Yui Shiiyama, Yuki Habu, Saeka Miyawaki, Chihiro Kaneko, Takuji Oyama, Isao Ishii

**Affiliations:** 1 Department of Health Chemistry, Showa Pharmaceutical University, Machida 194-8543, Tokyo, Japan; d2103@g.shoyaku.ac.jp (A.H.); kamata@ac.shoyaku.ac.jp (S.K.); kajiwara@ac.shoyaku.ac.jp (M.A.); b17112@ug.shoyaku.ac.jp (Y.M.); a17024@ug.shoyaku.ac.jp (K.U.); b16049@ug.shoyaku.ac.jp (Y.S.); b17096@ug.shoyaku.ac.jp (Y.H.); b18107@ug.shoyaku.ac.jp (S.M.); b18034@ug.shoyaku.ac.jp (C.K.); 2 Faculty of Life and Environmental Sciences, University of Yamanashi, Kofu 400-8510, Yamanashi, Japan; takujio@yamanashi.ac.jp

**Keywords:** bezafibrate, fenofibric acid, pemafibrate, peroxisome proliferator-activated receptor, dual/pan agonist, X-ray crystallography

## Abstract

Among the agonists against three peroxisome proliferator-activated receptor (PPAR) subtypes, those against PPARα (fibrates) and PPARγ (glitazones) are currently used to treat dyslipidemia and type 2 diabetes, respectively, whereas PPARδ agonists are expected to be the next-generation metabolic disease drug. In addition, some dual/pan PPAR agonists are currently being investigated via clinical trials as one of the first curative drugs against nonalcoholic fatty liver disease (NAFLD). Because PPARα/δ/γ share considerable amino acid identity and three-dimensional structures, especially in ligand-binding domains (LBDs), clinically approved fibrates, such as bezafibrate, fenofibric acid, and pemafibrate, could also act on PPARδ/γ when used as anti-NAFLD drugs. Therefore, this study examined their PPARα/δ/γ selectivity using three independent assays—a dual luciferase-based GAL4 transactivation assay for COS-7 cells, time-resolved fluorescence resonance energy transfer-based coactivator recruitment assay, and circular dichroism spectroscopy-based thermostability assay. Although the efficacy and efficiency highly varied between agonists, assay types, and PPAR subtypes, the three fibrates, except fenofibric acid that did not affect PPARδ-mediated transactivation and coactivator recruitment, activated all PPAR subtypes in those assays. Furthermore, we aimed to obtain cocrystal structures of PPARδ/γ-LBD and the three fibrates via X-ray diffraction and versatile crystallization methods, which we recently used to obtain 34 structures of PPARα-LBD cocrystallized with 17 ligands, including the fibrates. We herein reveal five novel high-resolution structures of PPARδ/γ–bezafibrate, PPARγ–fenofibric acid, and PPARδ/γ–pemafibrate, thereby providing the molecular basis for their application beyond dyslipidemia treatment.

## 1. Introduction

Peroxisome proliferator-activated receptors (PPARs) belong to the nuclear receptor (NR) superfamily and ligand-activated transcription factors that sense intracellular free fatty acids [1]. Three subtypes (PPARα, PPARβ/δ, and PPARγ) with considerable amino acid identity (54–71% in humans) have been identified in mammals. PPARα regulates lipid metabolism mainly in the liver and skeletal muscle and glucose homeostasis via direct transcriptional control of genes involved in peroxisomal/mitochondrial β-oxidation, fatty acid uptake, and triglyceride (TG) catabolism [2]. PPARδ is ubiquitously expressed and controls energy metabolism and cell survival [3]. PPARγ is most highly expressed in white/brown adipose tissues, where it acts as a master regulator of adipogenesis and a potent modulator of whole-body lipid metabolism and insulin sensitivity [4]. Three PPARs, similar to other NRs, comprise amino-terminal domains containing the activation function (AF)-1, DNA-binding domain, hinge region, and ligand-binding domain (LBD) containing the AF-2 region, and carboxyl-terminal domains [1]. Their ligand-binding pockets (LBPs) are relatively large, with a total volume of 1300–1400 Å^3^, compared to those found in other NRs that have 600–1100 Å^3^ LBPs [5]. Fatty acids and their derivatives from diet, de novo lipogenesis, and TG lipolysis are considered natural PPAR ligands [6,7]. Upon ligand binding, PPARs manifest conformational changes that facilitate corepressor molecule dissociation to enable the spatiotemporally orchestrated recruitment (association) of coactivators to the ligand-bound receptors [8]. Coactivators contain one or more highly conserved LXXLL α-helix motif called an NR box for direct interaction with AF-2 regions in PPARs [8].

Synthetic PPARα agonists “fibrates” have been widely used to treat hypertriglyceridemia; they decrease blood TG levels and increase high-density lipoprotein-cholesterol levels [9]. Bezafibrate, fenofibrate, ciprofibrate, clofibrate, and gemfibrozil were developed about half a century ago and have been clinically used in many countries [9]. Synthetic PPARγ agonists “thiazolidinediones (glitazones),” such as rosiglitazone and pioglitazone, are antidiabetic drugs with potent insulin-sensitizing effects that confer long-term glycemic control, although some of them might induce serious adverse effects, including edema, bone fracture, and heart failure [10]. PPARδ agonists are not yet clinically available but are expected to treat metabolic or cardiovascular diseases [11]. The development of a PPARδ-selective agonist seladelpar (MBX-8025) for non-alcoholic steatohepatitis (NASH) and primary sclerosing cholangitis (PSC) has been once discontinued at phase 2b clinical trial [12] and seladelpar is now only in phase 3 trial for primary biliary cholangitis (ClinicalTrials.gov number: NCT03301506) [13], although the Food and Drug Administration (FDA) lifted clinical holds on seladelpar for investigational new drug applications in NASH and PSC on 23 July 2020 [14]. Furthermore, PPAR dual/pan agonists are expected to treat metabolic diseases including non-alcoholic fatty liver disease (NAFLD) and NASH [15]. Saroglitazar (α/γ dual) and lanifibranor (α/δ/γ pan) are currently in clinical trials [16,17]. However, the development of most PPARα/γ dual agonists (e.g., muraglitazar, tesaglitazar, and aleglitazar) has been abandoned due to serious safety concerns [11], and that of a PPARα/δ dual agonist elafibranor for NASH has been discontinued due to it having no significant benefits [18]. Thus, the management of PPARα/δ/γ selectivity is indispensable.

We have recently revealed 34 novel high-resolution X-ray cocrystal structures of PPARα-LBD and 17 PPARα ligands, including bezafibrate, fenofibric acid, and pemafibrate, using sophisticated cocrystallization techniques [6,19] and further obtained PPARγ-LBD–saroglitazar structures [20]. The aim of this study was to use X-ray crystallography to examine the PPARα/δ/γ selectivity of clinically used fibrates in three independent assays and to provide its structural basis because PPARs have relatively large LBPs to accept 1–4 small molecule ligands [6]. Thus, this study demonstrated the PPAR dual/pan agonistic activities of all of those fibrates and revealed the novel five high-resolution structures of PPARδ/γ-LBD–bezafibrate, PPARγ-LBD–fenofibric acid, and PPARδ/γ-LBD–pemafibrate.

## 2. Results

### 2.1. Fibrates Induce Transactivation of Gene Expression via PPARα/δ/γ-LBD

**Figure 1:**
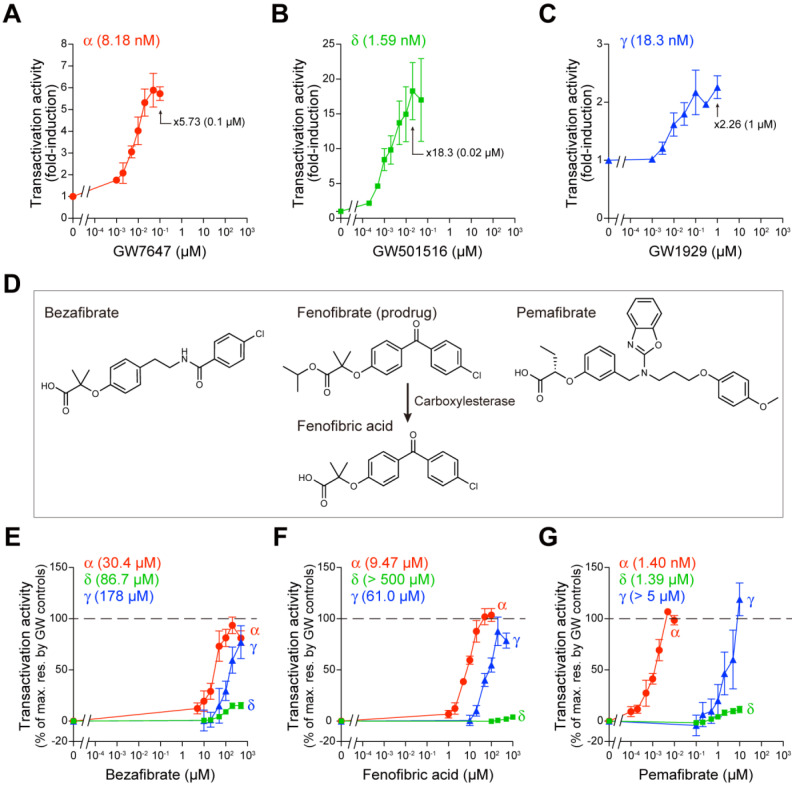
PPARα/δ/γ-LBD-mediated transactivation assay in COS-7 cells. (**A**–**C**) Control experiments. Human PPARα/δ/γ-LBD-mediated *Firefly* luciferase transactivation was induced by selective PPAR agonists: GW7647 for PPARα (**A**), GW501516 for PPARδ (**B**), and GW1929 for PPARγ (**C**) in a concentration-dependent manner. The maximal responses were observed at 0.1 µM, 0.02 µM, and 1 µM, respectively, which are used as the 100% responses in (**E**–**G**). (**D**) Chemical structures of fibrates used in this study. Fenofibrate is a prodrug that is metabolized by tissue and plasma carboxylesterases [22] to its active form, fenofibric acid. (**E**–**G**) PPARα/δ/γ-LBD-mediated transactivation by bezafibrate (**E**), fenofibric acid (**F**), and pemafibrate (**G**). Data are means ± standard error (SE) of three independent experiments with duplicate samples, and calculated EC_50_ values are shown.

The GAL4–hPPARα/δ/γ-LBD transactivation system [21] was employed to elucidate the impacts of fibrates on PPARα/δ/γ-LBD-mediated gene transcription activation (transactivation) in COS-7 cells. In this system, the *Firefly* luciferase reporter gene was activated only by the GAL4–human PPARα/δ/γ-LBD chimera, and the potentially confounding effects of endogenous receptors were eliminated. Transfection efficiency was normalized by *Renilla* luciferase activity. First, we confirmed that a potent PPARα-selective agonist GW7647 induced PPARα-LBD-mediated transactivation in a concentration-dependent manner with maximal effect (×5.73 of the basal activity) at 0.1 µM and an EC_50_ value of 8.18 nM (Figure 1A); a potent PPARδ-selective agonist GW501516 induced PPARδ-LBD-mediated transactivation with maximal effect (×18.3) at 0.02 µM and an EC_50_ of 1.59 nM (Figure 1B); and a potent PPARγ-selective agonist GW1929 induced PPARγ-LBD-mediated transactivation with maximal effect (×2.26) at 1 µM and an EC_50_ of 18.3 nM (Figure 1C).

We then compared the PPARα/δ/γ-LBD-mediated transactivation by the three fibrates—bezafibrate, fenofibric acid (an active metabolite of its prodrug fenofibrate [22]), and pemafibrate (Figure 1D)—considering the maximal effects by the GW compounds as 100%. Bezafibrate activated all PPAR subtypes, with 93.6% efficacy and 30.4 µM EC_50_ for PPARα, 15.2% efficacy and 86.7 µM EC_50_ for PPARδ, and 77.1% efficacy and 178 µM EC_50_ for PPARγ (Figure 1E). However, fenofibric acid activated PPARα (104% efficacy and 9.47 µM EC_50_) and PPARγ (87.7% efficacy and 61.0 µM EC_50_) but not PPARδ (Figure 1F). The concentrations at which pemafibrate (known as a selective PPAR modulator α “SPPARMα”) activated PPARα were three orders lower (107% efficacy and 1.40 nM EC_50_) than those at which it activated PPARδ (11.3% efficacy and 1.39 µM EC_50_) or PPARγ (119% efficacy and EC_50_ > 5 µM) (Figure 1G).

### 2.2. Fibrates Induce PPARγ Coactivator 1α (PGC1α) or Steroid Receptor Coactivator 1 (SRC1) Recruitment via PPARα/δ/γ-LBD

**Figure 2:**
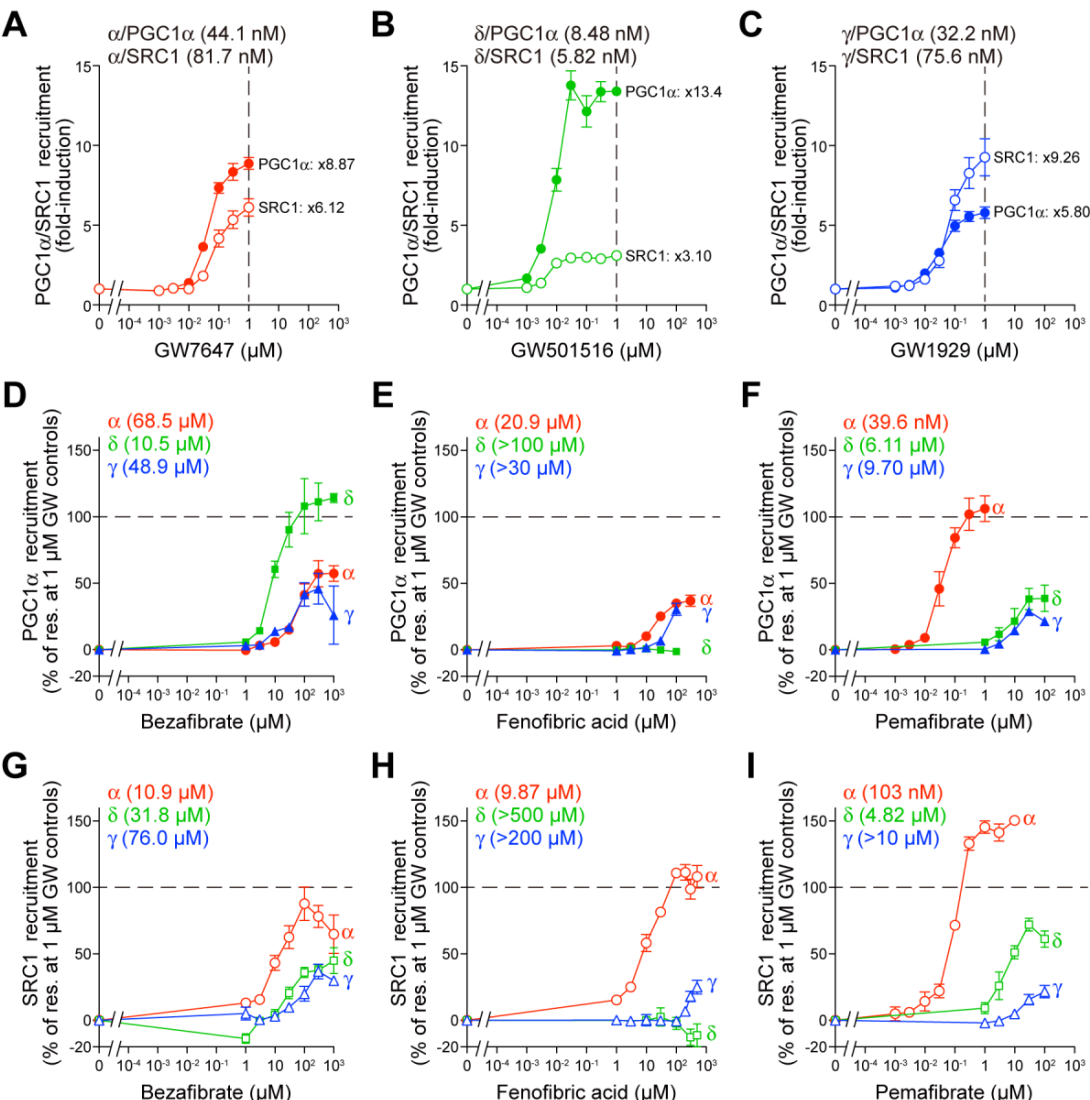
TR-FRET-based PPARα/δ/γ-LBD coactivator recruitment assay. (**A**–**C**) Control experiments. Human PPARα/δ/γ-LBD-mediated recruitment of coactivator peptides, PGC1α (filled symbols) and SRC1 (open symbols), was induced by selective PPAR agonists, GW7647 for PPARα (**A**), GW501516 for PPARδ (**B**), and GW1929 for PPARγ (**C**) in a concentration-dependent manner. Their maximal responses at 1 µM are used as the 100% responses in (**D**–**I**). (**D**–**I**) PPARα/δ/γ-LBD-mediated PGC1α (**D**–**F**) and SRC1 (**G**–**I**) coactivator peptide recruitment was induced by bezafibrate (**D**,**G**), fenofibric acid (**E**,H), and pemafibrate (**F**,**I**) in a concentration-dependent manner. Data are means ± SE of 3–4 independent experiments with duplicate samples, and calculated EC_50_ values are shown.

In the nucleus, PPARs remain largely in repressed states due to the presence of corepressors, such as nuclear receptor corepressor 1 (NCoR1) and NCoR2 (SMRT), bound to the *cis*-elements (PPREs: PPAR responsive elements) located in the promoter region of their multiple target genes, irrespective of their ligand binding status [8]. Ligand binding initiates a complicated transcription process, which includes the dissociation of the corepressor protein complexes and the association of coactivator protein complexes for linking to the basal transcription machinery [8]. The ligand-induced AF-2 helix 12 formation, which recruits coactivators such as PGC1α and SRC1, is a hallmark of PPAR activation. PGC1α has a preference for PPARα/γ and is highly expressed in brown adipose tissues and cardiac and skeletal muscles, whereas SRC1 has a preference for all PPAR subtypes and is highly expressed in brown and white adipose tissues and the brain [8]. Thus, time-resolved fluorescence resonance energy transfer (TR-FRET)-based detection of the physical association between PPARα/δ/γ-LBD and coactivators becomes a highly sensitive cell-free assay for evaluating PPAR ligand activities. First, we confirmed that GW7647 activates the recruitment of both coactivators in a concentration-dependent manner, with maximal effect (×8.87 of the basal activity) at 1 µM and 44.1 nM EC_50_ for PGC1α and ×6.12 at 1 µM and 81.7 nM EC_50_ for SRC1 (Figure 2A). GW501516 induced a maximal effect of ×13.4 at 1 µM with 8.48 nM EC_50_ for PGC1α and ×3.10 at 1 µM with 5.82 nM EC_50_ for SRC1 (Figure 2B), whereas GW1929 induced a maximal effect of ×5.80 at 1 µM with 32.2 nM EC_50_ for PGC1α and ×9.26 at 1 µM with 75.6 nM EC_50_ for SRC1 (Figure 2C). Next, we again compared the PGC1α/SRC1 recruitment activity of the three fibrates considering the maximal effects by the GW compounds as 100%. Bezafibrate and pemafibrate recruited the PGC1α peptide to all PPARα/δ/γ-LBD; however, fenofibric acid did not recruit PGC1α to PPARδ-LBD (Figure 2D–F). The situation was the same for SRC1 recruitment in that the efficacy and efficiency varied greatly between agonists, PPAR subtypes, and coactivator species (Figure 2G–I).

### 2.3. Fibrates Induce the Thermostability of PPARα/δ/γ-LBD

**Figure 3:**
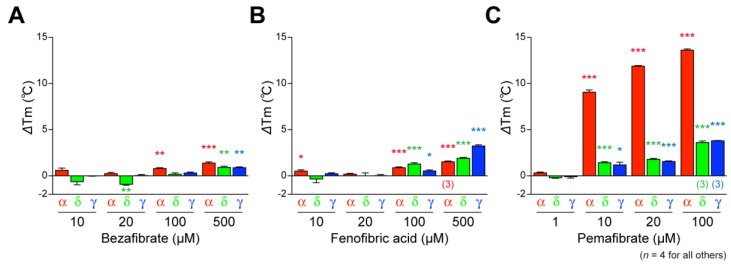
Circular dichroism-based PPARα/δ/γ-LBD thermostability assay. Bezafibrate—(**A**), fenofibric acid—(**B**), and pemafibrate-dependent (**C**) increases in *T*_m_ values (∆*T*_m_) at 222 nm were measured as the reflection of the thermostability of α-helical structures in PPARα/δ/γ-LBD. Data are means ± SE of four independent experiments (*n* = 3 for 500 µM fenofibric acid on PPARα-LBD and 100 µM pemafibrate on PPARδ/γ-LBD). Differences versus basal (0.1% DMSO) levels are significant in * *p*< 0.05, ** *p*< 0.01, and *** *p*< 0.001 in Student’s *t*-test.

PPARs show increased thermostability upon ligand binding, which can be detected using circular dichroism (CD) spectroscopy [6]. Ligand-induced alterations in *T*_m_ values at 222 nm were investigated as types of reflection of α-helical stable structures of PPARs because PPAR ligand binding induces stabilization of the LBP [23,24]. The basal (solvent [0.1% DMSO] only) *T*_m_ values were 49.54 °C (±0.12 °C) (*n* = 4), 51.76 °C (±0.17 °C) (*n* = 6), and 48.95 °C (±0.16 °C) (*n* = 4) for PPARα-LBD, PPARδ-LBD, and PPARγ-LBD, respectively, and interestingly, all the three fibrates increased *T*_m_ values of all PPAR subtypes, although the efficacy and efficiency varied significantly (Figure 3A–C).

### 2.4. Structures of the PPARα/δ/γ-LBD–Bezafibrate Complexes

**Figure 4:**
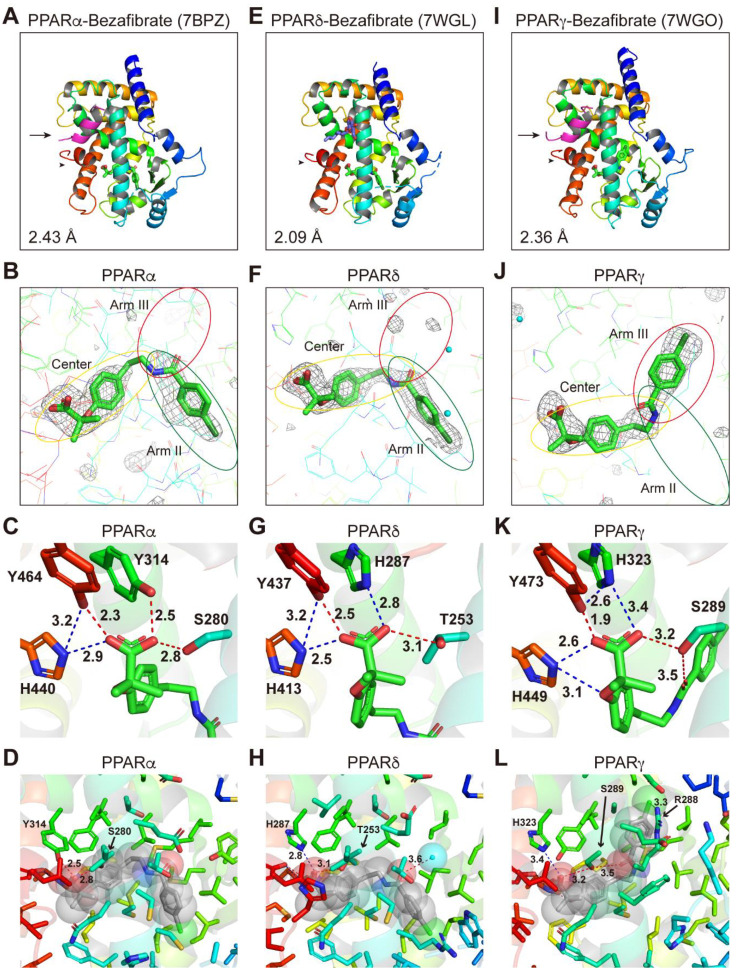
PPARα/δ/γ-LBD–bezafibrate cocrystal structures. Cocrystals of bezafibrate and PPARα-LBD (**A**–**D**; reprinted from Kamata et al. [6]), PPARδ-LBD (**E**–**H**), or PPARγ-LBD (**I**–**L**) were analyzed using X-ray diffraction. (**A**,**E**,**I**) Overall structures of the complexes. The SRC1 peptide (α-helix in magenta) and the AF-2 helix 12 (α-helix in red) are indicated by arrows and arrowheads, respectively. PDB identities and resolutions are labeled. (**B**,**F**,**J**) Magnified views of bezafibrate located in the Center/Arm II regions of PPARα/δ-LBD (**B**,**F**) or the Center/Arm III regions of PPARγ-LBD (**J**). The electron density is shown in the mesh via *F*_o_-*F*_c_ omit maps contoured at +3.0σ. Water molecules are presented as cyan spheres. (**C**,**G**,**K**) Hydrogen bonds and electrostatic interactions between bezafibrate and the four consensus amino acid residues (that recognize the carboxyl moiety of bezafibrate) are indicated by red and blue dotted lines, respectively, with their distances (Å). (**D**,**H**,**L**) Hydrogen bonds and electrostatic interactions between bezafibrate (in van der Waals spheres) and all surrounding amino acid residues located within a distance of 5 Å.

We have recently revealed the structures of 34 PPARα-LBD complexed with 17 ligands, including the three fibrates [6] and the structure of PPARγ-LBD–saroglitazar (a PPARα/γ dual agonist in clinical trials for NAFLD treatment) [20]. To gain structural insight into the PPARα/δ/γ selectivity of the fibrates, we aimed to obtain the structures of PPARδ/γ–fibrate complexes by X-ray crystallography and compare them with PPARα complex structures that we obtained (Figure 4A–D; reprinted from Kamata et al. [6]). We first screened various cocrystallization buffer conditions based on previous literature and our experience with PPARα/γ [19] (see Materials and Methods). A PPARδ-LBD–bezafibrate structure was obtained without using any coactivators (Figure 4E–H), whereas PPARγ-LBD–bezafibrate cocrystals were obtained using the SRC1 peptide (Figure 4I–L; SRC1 is indicated by an arrow in Figure 4I). The complex structures of bezafibrate bound to PPARδ/γ-LBD were solved in a monoclinic space group *P*2_1_ at 2.09 Å resolution (Figure 4E; deposited in the Protein Data Bank (PDB) with ID: 7WGL) and an orthorhombic space group *P*2_1_2_1_2_1_ at 2.36 Å resolution (PDB ID: 7WGO), respectively (Supplementary Table S1). The electron density map for bezafibrate in all PPARα/δ/γ-LBD indicated the presence of a single molecule in the protein monomer (Figure 4B,F,J).

The overall structures were identical to the previously reported active conformations that form the AF-2 helix 12, which provided root mean square (RMS) distances of 0.57 Å (219 common Cα positions in PPARα/δ) and 0.61 Å (218 common Cα positions in PPARα/γ) (Figure 4A,E,I). We have previously defined five LBPs (Arm I–III/X and Center) in PPARα-LBD based on our and others’ observations of complexes with a total of 38 ligands [19], and four similar pockets (Arm I–III/X) in PPARδ/γ-LBD [20]. The chlorobenzyl moiety of bezafibrate was located in the Arm II region of PPARδ-LBD (Figure 4F) similar to that observed in PPARα-LBD (Figure 4B) [6]; however, it was rotated at an angle of 136.8° to reside in the Arm III region of PPARγ-LBD (Figure 4J). The carboxylic groups of the fibrates may stabilize the AF-2 helix 12 formation through hydrogen bonds (red dotted lines) and electrostatic interactions (blue dotted lines) with the four consensus amino acids, PPARα, Ser280/Tyr314/His440/Tyr464 (Figure 4C) [6]; PPARδ, Thr253/His287/His413/Tyr437 (Figure 4G); and PPARγ, Ser289/His323/His449/Tyr473 (Figure 4K), although a very close proximity (2.3 Å or 1.9 Å < 2.4 Å) was observed in PPARα Y464 (Figure 4C) or PPARγ Y473 (Figure 4K). Hydrogen bonds were also observed between bezafibrate and a water molecule (3.6 Å; Figure 4H) and between PPARγ S289 and the carbonyl group of bezafibrate (3.5 Å; Figure 4L). A single halogen bond was observed between PPARγ R288 and the chlorine atom of bezafibrate (3.3 Å; Figure 4L). Those interactions in PPARγ might stabilize the rotated chlorobenzyl moiety of bezafibrate.

When amino acid residues in the PPARα/δ/γ-LBD are colored by their hydrophobicity (red) and hydrophilicity (white) using a Color h script program [25], all three fibrates (illustrated by their van der Waals spheres) were mainly surrounded by hydrophobic residues of amino acids in PPARα/δ/γ-LBD (Supplementary Figure S1).

### 2.5. Structures of the PPARα/γ-LBD–Fenofibric Acid Complexes

**Figure 5:**
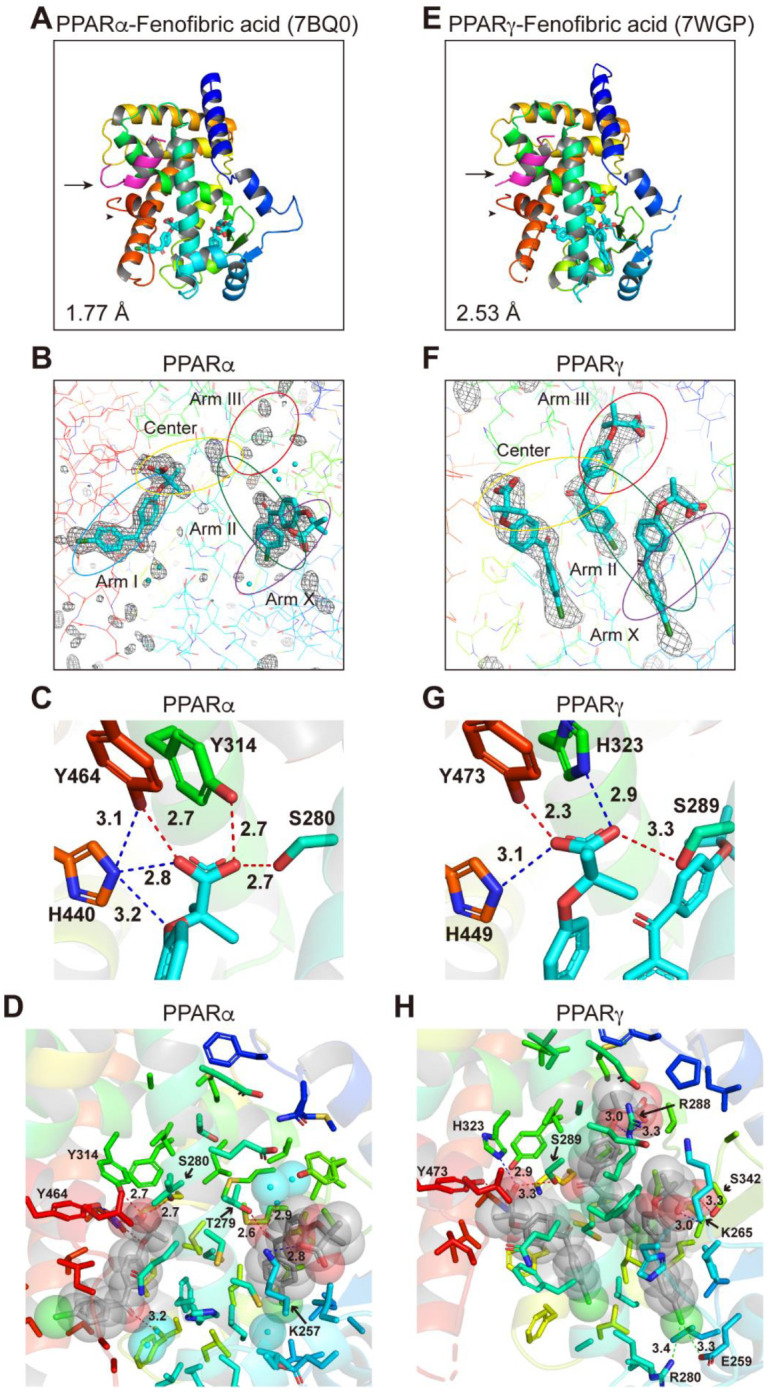
PPARα/γ-LBD–fenofibric acid cocrystal structures. Cocrystals of fenofibric acid and PPARα-LBD (**A**–**D**; reprinted from Kamata et al. [6]) or PPARγ-LBD (**E**–**H**) were analyzed using X-ray diffraction. (**A**,**E**) Overall structures of the complexes. The SRC1 peptide (α-helix in magenta) and the AF-2 helix 12 (α-helix in red) are indicated by arrows and arrowheads, respectively. PDB identities and resolutions are labeled. (**B**,**F**) Magnified views of fenofibric acids located in Arm I/Center regions (single molecule) and Arm II/Arm X regions (another) of PPARα-LBD (**B**) or the Center region (1st molecule), Arm II/Arm III regions (2nd), and Arm II/Arm X (3rd) of PPARγ-LBD (**F**). The electron density is shown in the mesh via *F*_o_-*F*_c_ omit maps contoured at +3.0σ. Water molecules are presented as cyan spheres. (**C**,**G**)Hydrogen bonds and electrostatic interactions between fenofibric acid and the four consensus amino acid residues (that recognize the carboxyl moiety of fenofibric acid) are indicated by red and blue dotted lines, with their distances (Å). (**D**,**H**) Hydrogen bonds and electrostatic interactions between fenofibric acid (in van der Waals spheres) and all surrounding amino acid residues located within a distance of 5 Å.

Cocrystals of PPARδ-LBD–fenofibric acid were not obtained probably because of its low binding affinity (Figure 1F and Figure 2E,H), although increased thermostability was observed at high concentrations (Figure 3B). However, cocrystals with PPARγ-LBD were obtained in the presence of the SRC1 peptide. The complex structure was resolved in the orthorhombic space group *P*2_1_2_1_2_1_ at 2.53 Å resolution (PDB ID: 7WGP) (Supplementary Table S1). The electron density map for fenofibric acid bound to PPARα-LBD indicated the presence of two molecules in the protein monomer (Figure 5A–D; reprinted from Kamata et al. [19]); however, that of fenofibric acid bound to PPARγ-LBD indicated the existence of three molecules in the protein monomer (Figure 5E–H). Its overall structure was basically identical to the previously reported active conformations that form the AF-2 helix 12 (arrowheads in Figure 5A,E). The two structures were similar, with an RMS distance of 0.57 Å (215 common Cα positions). 

In PPARα-LBD, two molecules were located at the Center/Arm I and Arm II/X (Figure 5A,B). In PPARγ-LBD, the first molecule was located beside the Center region, the second molecule at Arm II/III, and the third molecule at Arm II/X (Figure 5F). Only the first molecule was stabilized by the four consensus amino acids (Ser289/His323/His449/Tyr473) via hydrogen bonds *(*red dotted lines) and electrostatic interactions (blue dotted lines) in PPARγ-LBD (Figure 5G). Hydrogen bonds were also observed between PPARα K257 and the carbonyl group of the first molecule (water-mediated) and between PPARα T279 and the carbonyl group of the second molecule (Figure 5D). No hydrogen bond or electrostatic interaction was observed between PPARγ and the first molecule, but hydrogen bonds were observed between PPARγ R288 and the oxygen atom of the second molecule and between PPARγ S342 and the carboxylic acid of the third molecule. Furthermore, two halogen bonds were observed between PPARγ E259/R280 and the chlorine atom of the third molecule (Figure 5H). Such interactions combined with hydrophobic interactions (Supplementary Figure S1D,E) may stabilize the location of the second and third fenofibric acid molecules.

### 2.6. Structures of the PPARα/δ/γ-LBD–Pemafibrate Complexes

**Figure 6:**
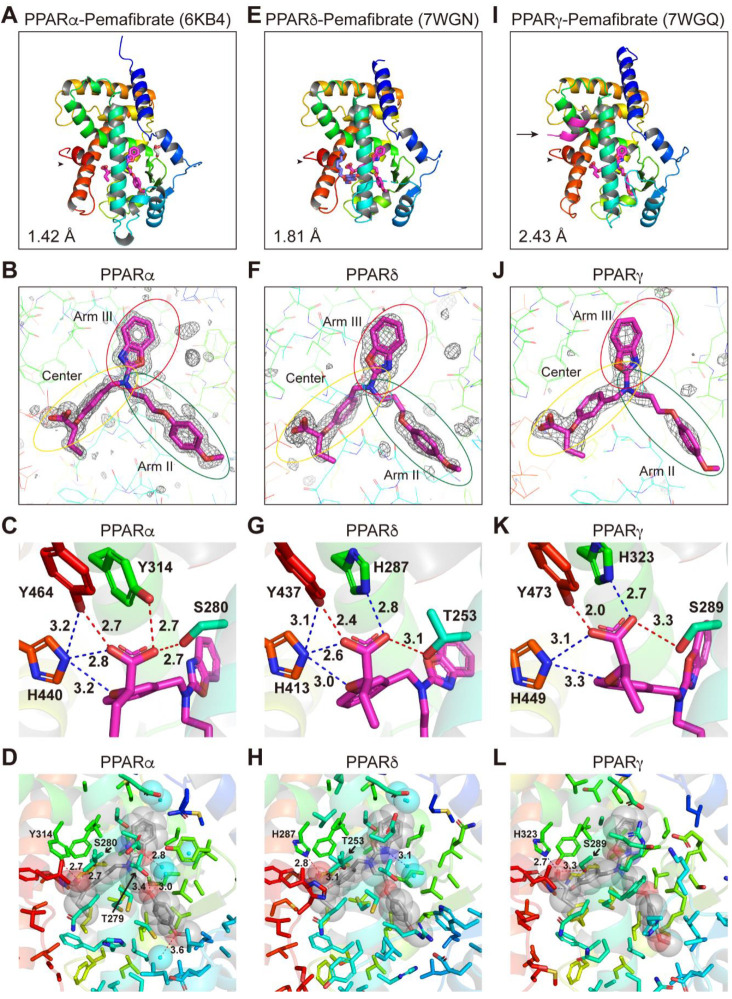
PPARα/δ/γ-LBD–pemafibrate cocrystal structures. Cocrystals of pemafibrate and PPARα-LBD (**A**–**D**; reprinted from Kamata et al. [6]), PPARδ-LBD (**E**–**H**), or PPARγ-LBD (**I**–**L**) were analyzed using X-ray diffraction. (**A**,**E**,**I**) Overall structures of the complexes. The SRC1 peptide (α-helix in magenta) and the AF-2 helix 12 (α-helix in red) are indicated by arrows and arrowheads, respectively. PDB identities and resolutions are labeled. (**B**,**F**,**J**) Magnified views of pemafibrate located in the Center/Arm II/Arm III regions of PPARα/δ/γ-LBD. The electron density is shown in the mesh via *F*_o_-*F*_c_ omit maps contoured at +3.0σ. Water molecules are presented as cyan spheres. (**C**,**G**,**K**) Hydrogen bonds and electrostatic interactions between pemafibrate and the four consensus amino acid residues (that recognize the carboxyl moiety of pemafibrate) are indicated by red and blue dotted lines, respectively, with their distances (Å). (**D**,**H**,**L**) Hydrogen bonds and electrostatic interactions between pemafibrate (in van der Waals spheres) and all surrounding amino acid residues located within a distance of 5 Å.

The PPARδ-LBD–pemafibrate cocrystals, similar to the PPARα-LBD–pemafibrate cocrystals (Figure 6A–D; reprinted from Kamata et al. [19]), were obtained without using coactivators (Figure 6E–H), whereas the PPARγ-LBD–pemafibrate cocrystals were obtained only using SRC1 (Figure 6I–L), suggesting that its physical interaction with PPARγ-LBD is the weakest (Figure 2F,I). Their structures were solved in the orthorhombic space group *P*22_1_2_1_ at 1.81 Å resolution (PDB ID: 7WGN) and the orthorhombic space group *P*2_1_2_1_2_1_ at 2.43 Å resolution (PDB ID: 7WGQ), respectively (Supplementary Table S1). The electron density of pemafibrate in PPARδ/γ-LBD indicated a single molecule in the protein monomer (Figure 6E,I) similar to that observed in PPARα-LBD (Figure 6A). Their overall structures were identical to the active conformations that form the AF-2 helix 12 (arrowheads in Figure 6A,E,I), with RMS distances of 0.75 Å (234 common Cα positions in PPARα/δ) and 0.71 Å (226 common Cα positions in PPARα/γ).

Pemafibrate was located at the almost identical Y-shaped structures comprising the Center and Arm II/III regions in all PPARs (Figure 6B,F,J) although its phenoxyalkyl group seemed to be pushed toward helix 5 in PPARδ-LBD (Figure 6E) and its 2-aminobenzoxazole group seemed to be pushed toward helix 3 in PPARγ-LBD (Figure 6I). Several hydrogen bonds and electrostatic interactions with the four consensus amino acids were observed (Figure 6C,G,K) with a very close proximity (2.0 Å) to PPARγ Y473 (Figure 6K). Hydrogen bonds between PPARα T279 and water molecules were also observed in PPARα (Figure 6D), whereas a water-mediated hydrogen bond or electrostatic interaction with 2-aminobenzoxazole group of pemafibrate was observed in PPARδ-LBD (Figure 6H). Furthermore, no such bonds or interactions were observed in PPARγ-LBD (Figure 6L). Pemafibrate was further stabilized by hydrophobic interactions in all PPARs (Supplementary Figure S1F–H).

### 2.7. Various Binding Modes to PPARα/δ/γ-LBD Pockets

**Figure 7:**
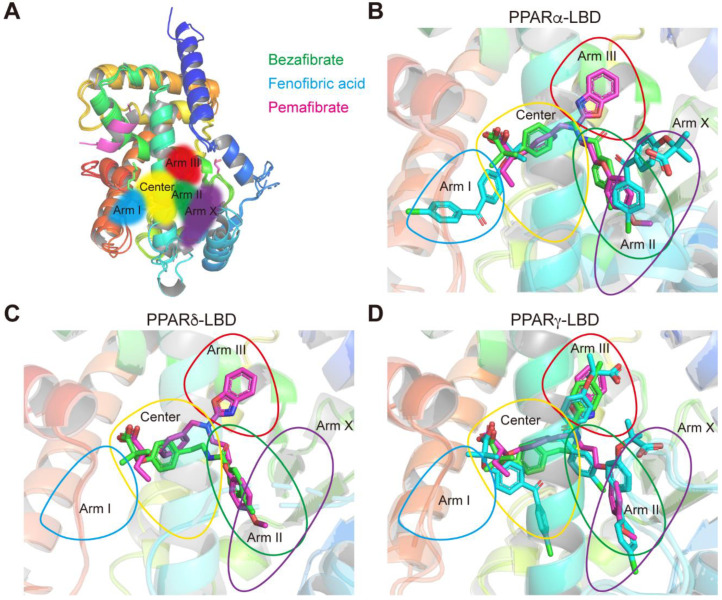
Ligand-binding pocket (LBP) regional localization of the three fibrates in PPARα/δ/γ-LBD. (**A**) LBP comprising the Center and Arms I–III and X of PPARα/δ/γ-LBD [6,17]. (**B**–**D**) Superimposed images of bezafibrate (green), fenofibric acid (blue), and pemafibrate (red) in PPARα/δ/γ-LBD.

We have previously defined five LBPs (Center and Arm I–III/X) in PPARα-LBD [19] (Figure 7A) and four LBPs (Center and Arm I–III) in PPARδ/γ-LBD [20]. Bezafibrate was located at the Center/Arm II regions of PPARα-LBD (Figure 7B), where endogenous fatty acids and many other ligands bind [19]. However, one fenofibric acid molecule was located at the Arm I and another was located at the Arm X of PPARα-LBD (Figure 7B); only the Arm I pocket is known to be occupied by relatively low affinity fibrates, such as ciprofibrate, clofibric acid, and gemfibrozil [19]. Pemafibrate was located at the Y-shape structures comprising the Center and Arm II/III (Figure 7B), similar to GW7647, the potent PPARα-selective agonist [19]. 

In PPARδ-LBD, bezafibrate was located at the Center/Arm II regions (Figure 7C), where the potent PPARδ-selective agonists such as GW501516 and GW0742 bind [26,27], and only pemafibrate was located at the Arm III region (Figure 7C) as in PPARα-LBD (Figure 7B). In PPARγ-LBD, a single bezafibrate was located at the Center/Arm III regions; three fenofibric acids were arranged in a parallel fashion within the Center and Arm II/III/X; and a single pemafibrate was in the Y-shape structures comprising the Center and Arm II/III (Figure 7D). Notably, bezafibrate and fenofibric acid (the second molecule) were located in the Arm III regions only in PPARγ-LBD (Figure 7D).

## 3. Discussion

Most fibrates were developed in the 1960s–1980s before their molecular target, PPARα, was identified. Therefore, neither the information regarding PPAR agonistic activity (including subtype selectivity) nor the results of molecular docking studies on the three-dimensional structures of PPARs were employed for designing and developing the fibrates. Classical fibrates, such as bezafibrate and fenofibrate, are known to have relatively low PPAR activity and selectivity [28]. Bezafibrate was launched in 1978 by Boehringer Mannheim in Germany (current F. Hoffmann-La Roche AG [Roche], Switzerland) and is currently approved in many countries but not the United States. It is considered to be a “balanced” pan agonist that activates all PPARα/δ/γ at comparable doses and improves dyslipidemia via its actions against PPARα; insulin sensitivity via its actions against PPARγ, and overweightness by enhancing fatty acid oxidation, energy consumption, and adaptive thermogenesis via its actions against PPARδ [29]. Bezafibrate activated all PPARα/δ/γ in the cell-based transactivation assay (Figure 1E), cell-free (both PGC1α and SRC1) coactivator recruitment assay (Figure 2D,G), and cell-free thermostability assay (Figure 3A) by binding to the similar binding pockets (Center/Arm II) of PPARα/δ (Figure 4B,F) or the other binding pocket (Center/Arm III) of PPARγ (Figure 4J). Interestingly, depending on the assays, bezafibrate induced responses with altered affinity against PPARα/δ/γ: the orders of EC_50_ values were α (30.4 µM) < δ (86.7 µM) < γ (178 µM) in transactivation (Figure 1E; equivalent to the values [α (25 µM); δ (95 µM); γ (>100 µM)] in previous transactivation experiments [30]), δ < γ < α in PGC1α recruitment (Figure 2D), α < δ < γ in SRC1 recruitment (Figure 2G), and α < δ = γ in thermostability assay (Figure 3A). In addition, bezafibrate exhibited altered efficacy: the orders of the maximal responses were α > γ > δ in transactivation (Figure 1E), δ > α > γ in PGC1α recruitment (Figure 2D), α > δ > γ in SRC1 recruitment (Figure 2G), and α > δ = γ in the thermostability assay (Figure 3A). In the phase 3 study of “Bezafibrate in Combination with Ursodeoxycholic Acid in Primary Biliary Cirrhosis (BEZURSO; NCT01654731)” completed in December 2016, its dual actions on PPARα/δ were considered important for the improvement of biochemical measures, such as liver stiffness [31]. However, the action of bezafibrate on PPARγ could also be indispensable for the effect. Another phase 2 study investigating the effect of bezafibrate on bipolar depression (NCT02481245) depended on the concept that mitochondrial dysregulation is attributable to bipolar depression, which could be ameliorated by PPAR pan agonists, such as bezafibrate.

Fenofibrate was introduced into clinical practice in 1974 and launched in France in 1975 [32]. More than 200 clinical trials on fenofibrate have been completed and are ongoing, and it has been approved in the United States and many other countries. Unlike bezafibrate, fenofibric acid has been recognized as a PPARα-selective agonist in clinical settings, although it was shown to activate PPARγ in several in vitro experiments [30,33]. Fenofibric acid induced PPARα/γ-LBD-mediated transactivation (Figure 1F), PGC1α/SRC1 recruitment (Figure 2E,H), increases in thermostability (Figure 3B), and bound to PPARα/γ-LBD (Figure 5). Interestingly, fenofibric acid did not induce transactivation (Figure 1F) and PGC1α/SRC1 recruitment (Figure 2E,H) but increased thermostability (Figure 3B) of PPARδ-LBD, and its cocrystals with PPARδ-LBD were not obtained. As observed in the thermostability assay (Figure 3B), fenofibric acid might bind to the non-LBP regions of PPARδ-LBD and stabilize the PPARδ-LBD but fail to functionally activate it. Unlike bezafibrate, fenofibric acid displayed a consistent order (α > γ >> δ = no response) of affinity and efficacy in transactivation (Figure 1F) and PGC1α/SRC1 recruitment (Figure 2E,H), except for thermostability (Figure 3B). To our knowledge, no publication has reported EC_50_ values of fenofibric acid or fenofibrate in PPARδ(-LBD)-mediated reactions; therefore, fenofibric acid is considered a genuine PPARα/γ dual agonist. Pemafibrate (K-877) was approved in 2018 in Japan as a highly selective PPARα agonist that is safe for simultaneous use with statins even in patients with mild adverse effects [34]. Pemafibrate activated PPARα/δ/γ-LBD-mediated transactivation (Figure 1G), PGC1α/SRC1 recruitment (Figure 2F,I), increases in thermostability (Figure 3C), and bound to PPARα/δ/γ-LBD with the similar Y-shaped forms (Figure 6), although it acted at much lower concentrations on PPARα-LBD than on PPARδ/γ-LBD. 

This study demonstrated that bezafibrate and pemafibrate could bind to and activate all PPAR subtypes, whereas fenofibric acid could bind to and activate only PPARα/γ. Whether this may happen in clinical settings is an issue of importance and needs to be investigated. Therapeutic doses in Japan are 800 mg, 106.6–160 mg, and 0.2 mg per day for bezafibrate, fenofibrate, and pemafibrate, respectively. The maximal plasma concentration (*C*_max_) after the administration of a single dose (200 mg) of bezafibrate was 20.4 µM (7.39 µg/mL) [35], which is roughly equivalent to its EC_50_ values in transactivation (Figure 1E) and PGC1α/SRC1 recruitment (Figure 2D,G); thus, bezafibrate can act as a PPAR pan activator in its clinical doses. According to the package insert of TRICOR (Takeda, Tokyo, Japan), the *C*_max_ after the administration of a single one-day dose (160 mg) of fenofibrate was 37.0 µM (11.8 µg/mL). Fenofibric acid (30 µM) activated PPARα and slightly activated PPARγ but did not activate PPARδ (Figure 1F and Figure 2E,H). Therefore, fenofibric acid is considered a relatively weak PPARα agonist. According to the package insert of PARMODIA (Kowa, Nagoya, Japan), the *C*_max_ after the administration of a single one-day maximal dose (400 µg in Japan) of pemafibrate was 7.28 nM (3.57 ng/mL) after seven-day repeats. Pemafibrate (7 nM) induced transactivation only in PPARα (Figure 1G); and the coactivator recruitment assay hardly detected signals produced by <10 nM ligands owing to too many non-responding PPAR-LBDs within a total of 100 or 200 nM proteins. Therefore, pemafibrate is considered a selective agonist of PPARα at its clinical doses.

The global prevalence of NAFLD is estimated to be 25% of the global population, which is consistent with the substantial increases in the number of patients with diabetes and metabolic syndrome. PPAR dual/pan agonists are expected to be some of the most promising therapeutic drugs for NAFLD [36]. Although saroglitazar (α/γ dual agonist; Zydus Discovery, Dubai, UAE) [37] and lanifibranor (α/δ/γ pan agonist; Iventiva Pharma, Daix, France) [17] remain under clinical trials investigating their use against NAFLD/NASH, the development of most of the dual PPARα/γ agonists (mostly against type 2 diabetes)— muraglitazar (Bristol-Myers Squibb/Merck), tesaglitazar (AstraZeneca), aleglitazar (Roche), MK0767 (Kyorin/Banyu/Merck), naveglitazar (Ligand Pharmaceuticals, San Diego, CA, USA), ONO-5219 (Ono Pharma, Osaka, Japan), and DSP-8658 (Sumitomo Dainippon Pharma, Osaka, Japan)—has been discontinued due to PPARγ-related side effects (e.g., heart failure) or no effects [11,38]. Therefore, repositioning of bezafibrate [39] and fenofibrate, which have proven safety, as anti-NAFLD/NASH drugs might be a favorable option. Although three clinical trials investigating the use of fenofibrate against NAFLD/NASH (NCT02781584, NCT00262964, NCT02891408) and a single trial investigating pemafibrate (NCT03350165) have failed, there have been no clinical trials on the use of bezafibrate against NAFLD/NASH. However, bezafibrate has been shown to exert beneficial effects on NASH in tamoxifen- or its analog toremifene-treated breast cancer patients [40,41]. Pemafibrate improved the FibroScan–aspartate aminotransferase (FAST) scores (a novel index of NASH conditions) [42] and the similar scores [43] in NAFLD/NASH patients in some retrospective studies. The differential (in terms of efficiency and efficacy) recruitment of coactivators (PGC1α and SRC1) by bezafibrate (Figure 2D,G) may induce the expression of the specific sets of genes different from those induced by fenofibric acid or pemafibrate [44] in organ-specific manners [45].

In conclusion, this study has highlighted the PPAR dual/pan agonistic aspects of the three approved fibrates—bezafibrate, fenofibric acid, and pemafibrate—using functional and structural analyses. PPAR dual/pan agonists could be a radical remedy for NAFLD/NASH, and our findings contribute toward the fine-tuning of PPAR subtype selectivity.

## 4. Materials and Methods

### 4.1. PPAR Activation Assay 1: Transactivation Assay

COS-7 cells (No. RCB0539; Riken BRC Cell Bank, Ibaraki, Japan) were maintained in Dulbecco’s modified Eagle’s medium (DMEM) supplemented with 10% fetal bovine serum (FBS) and antibiotics at 37 °C in a 5% CO_2_/95% air incubator. To evaluate PPARα/δ/γ-mediated transcriptional activation, pSG5-GAL–human PPARα/δ/γ chimera expression plasmids, a MH100(UAS)×4-tk-Luc reporter plasmid, and a pRL-CMV *Renilla* luciferase control plasmid under the control of a cytomegalovirus promoter were cotransfected into COS-7 cells. The pSG5-GAL–hPPARα/δ/γ plasmids express fusion proteins comprising the yeast transcription factor GAL4 DNA-binding domain and each of the human PPARα/δ/γ-LBDs [21,24]. The MH100(UAS)×4-tk-Luc plasmid contains four copies of MH100 GAL4 binding site and the *Firefly* luciferase gene [46]. The cells were seeded on 96-well tissue culture plates at a density of 1.0 × 10^4^ per well in 90 µL of DMEM supplemented with 1% FBS. After 24 h, 10 µL mixture containing 20 ng of pSG5-GAL-hPPARα/δ/γ, 80 ng of MH100(UAS)×4-tk-Luc, 30 ng of pRL-CMV, and 0.6 µL ViaFect transfection reagent (Promega, Madison, WI, USA) in Opti-MEM I reduced serum media (Thermo Fisher Scientific, Waltham, MA, USA) were added to each well. After 38 h, cells were treated with PPAR ligands (dissolved in 25 µL of DMEM with no FBS) and cultured for 10 h. Both *Firefly* and *Renilla* luciferase activities were measured using the Dual-Glo Luciferase Assay System (Promega). The transactivation activities were expressed as percentages of the maximal *Firefly* luciferase responses induced by potent/specific PPARα/δ/γ agonists: GW7647 (0.1 µM) for PPARα, GW501516 (0.02 µM) for PPARδ, and GW1929 (1 µM) for PPARγ—after normalization with *Renilla* luciferase responses. GW7647, GW501516, and bezafibrate were purchased from Cayman Chemical (Ann Arbor, MI, USA). GW1929, pemafibrate, and fenofibric acid were purchased from Sigma-Aldrich, ChemScene (Monmouth Junction, NJ, USA), and FujiFilm-Wako (Osaka, Japan), respectively.

### 4.2. Recombinant PPARα/δ/γ-LBD Expression and Purification

Human PPARα-LBD (amino acids 200–468), PPARδ-LBD (amino acids 170–441), and PPARγ-LBD (amino acids 203–477 in isoform 1) were expressed as amino-terminal His-tagged proteins by the pET28a vector (Merck KGaA (Novagen), Darmstadt, Germany) in Rosetta (DE3) pLysS competent cells (Novagen) and purified using three-step chromatography as described in our PPARα-LBD preparation [6,19]. Transformed cells were cultured at 30 °C in an LB medium (with 15 µg/mL kanamycin and 34 µg/mL chloramphenicol), and 50 mL of overnight culture was seeded in 1 L of a TB medium (with 15 µg/mL kanamycin), which was cultured at 30 °C for 1.5 h and then at 15 °C for 2 h. Protein overexpression was induced by adding 0.5 mM isopropyl β-d-thiogalactopyranoside, which were later cultured at 15 °C for 48 h. The cells were harvested and resuspended in 40 mL of buffer A (20 mM Tris-HCl [pH 8.0], 150 mM NaCl, 1 mM Tris 2-carboxyethylphosphine [TCEP]-HCl, and 10% glycerol) for PPARα/γ or buffer A’ (20 mM Tris-HCl [pH 8.0], 500 mM ammonium acetate, 1 mM TCEP-HCl, and 10% glycerol) for PPARδ; both buffers contained a complete EDTA-free protease inhibitor (Sigma-Aldrich). The cells were then lysed by sonication five times, for 2 min each time, using a UD-201 sonicator (Tomy, Tokyo, Japan) at an output of eight; they were clarified by centrifugation at 12,000× *g* at 4 °C for 20 min (these conditions were used throughout the study unless otherwise noted); then, polyethyleneimine, at a final concentration of 0.15% (*v*/*v*), was added to the supernatant to remove nucleic acids. After centrifugation, 35 mL of the supernatant was mixed with 20 g of ammonium sulfate at 4 °C for 30 min using gentle rotation. After centrifugation, the pellet was resuspended in 30 mL of buffer B (for PPARα/γ) or B’ (for PPARδ); these buffers were based on buffer A or A’ supplemented with 10 mM imidazole, respectively. The suspension was loaded on a cobalt-based immobilized metal affinity column (TALON Metal Affinity Resin, Takara Bio (Clontech), Shiga, Japan) equilibrated with buffer B (or B’) and eluted with a linear gradient of 10–100 mM imidazole. The PPARα/δ/γ-LBD-containing elutes were incubated with 33 U/mL thrombin protease (Nacalai Tesque, Kyoto, Japan) to cleave the His-tag and simultaneously dialyzed against buffer A (or A’) overnight at 4 °C using a Slide-A-Lyzer G2 dialysis cassette (20 kDa cutoff, Thermo Fisher Scientific). The sample was later dialyzed against buffer C, which was buffer A without 150 mM NaCl, at 4 °C for 3 h. The sample was then loaded onto a HiTrap Q anion-exchange column (GE Healthcare, Boston, MA, USA) equilibrated with buffer C, and eluted with a linear gradient of 0–150 mM NaCl (or 0–0.5 M ammonium acetate for PPARδ). The elutes were loaded onto a HiLoad 16/600 Superdex 75 pg gel-filtration column (GE Healthcare), which had been equilibrated with buffer A (or A’) and further eluted with buffer A (or A’).

### 4.3. PPAR Activation Assay 2: PGC1α/SRC1 Coactivator Recruitment Assay

The activation status of each PPARα/δ/γ subtype can also be determined using a TR-FRET assay, which is used to detect physical interactions between His-tagged hPPARα/δ/γ-LBD proteins and a biotin-labeled PGC1α coactivator peptide (biotin-EAEEPSLLKKLLLAPANTQ [amino acids 137–155] synthesized by GenScript) or SRC1 peptide (biotin-CPSSHSSLTERHKILHRLLQEGSPS [amino acids 676–700] from GenScript) using the LANCE Ultra TR-FRET assay (PerkinElmer) [6]. A 9.5 µL aliquot of PPARα/δ/γ-LBD (200 nM in buffer D for PPARα/γ-LBD or 400 nM in buffer E for PPARδ-LBD), 0.5 µL of a 100× ligand solution (in DMSO), and 5 µL of biotin-PGC1α (4 µM) or biotin-SRC1 peptide (8 µM) were mixed in one well of a Corning 384-well low-volume, white-round-bottom, polystyrene non-binding surface microplate (buffer D comprised 10 mM HEPES-NaOH [pH7.4], 150 mM NaCl, 0.005% Tween 20, 0.1% fatty acid-free bovine serum albumin [BSA]; buffer E comprised 50 mM HEPES-NaOH [pH7.4], 50 mM KCl, 1 mM EDTA, 0.5 mM dithiothreitol, and 0.1% fatty acid-free BSA). Then, 5 µL of 4 nM Eu-W1024-labeled anti-6×His antibody/80 nM ULight-Streptavidin (PerkinElmer) were added to each well, and the microplate was incubated in the dark for 2 h at room temperature. FRET signals were detected at one excitation filter (340/12) and at two emission filters (615/12 and 665/12) using a Varioskan Flash double monochromator microplate reader (Thermo Fisher Scientific). The parameters for the measurements at 615 nm and 665 nm were an integration time of 200 µs and a delay time of 100 µs. The 665 nm emissions were due to ULight-FRET, and the 615 nm emissions were due to Eu-W1024. The 665/615 ratio was calculated and normalized to the negative control reaction using 1% DMSO. The nonlinear fitting and calculation of EC_50_ were performed using Prism 5 software (GraphPad, San Diego, CA, USA).

### 4.4. PPAR Activation Assay 3: Thermostability Assay Using CD Spectroscopy

PPARα/δ/γ-LBD proteins (10 µM) were incubated with different concentrations of ligands in buffer A. CD spectra were monitored within 200–260 nm at increasing temperatures from 30 °C to 70 °C (2 °C/min) using a J-1500 spectropolarimeter equipped with a PTC-510 thermal controller (JASCO, Tokyo, Japan). The spectra of all PPARs displayed local minima at 208 nm and 222 nm (data not shown), a typical feature of α-helical proteins [47]. The thermal stability of PPARs was investigated by continuously monitoring the ellipticity changes at 222 nm during thermal denaturation [23,24], and a single-site sigmoidal dose-response curve fitting program (Prism 5) was used to obtain the melting temperature (*T*_m_) that corresponds to the midpoint of the denaturation process. The ligand-induced increases in *T*_m_ values are defined as ∆*T*_m_.

### 4.5. Cocrystallization of PPARδ/γ-LBD with the Three Fibrates

Cocrystallization of PPARδ-LBD was performed in hanging-drop mixtures of 0.5 µL of PPARδ-LBD (10 mg/mL in buffer A’), 0.1 µL of 10 mM ligand, 0.3 µL of buffer A’, 0.1 µL of 5% *n*-octyl-β-d-glucoside, and 1 µL reservoir solution (50 mM Bis-Tris propane [pH 8.5], 14% PEG8000, 0.2 M KCl, 6% propanediol, 1 mM EDTA, 1 mM CaCl_2_ for PPARδ-LBD/pemafibrate; 50 mM Bis-Tris propane [pH 8.5], 14% PEG8000, 0.1 M KSCN, 6% propanediol, 1 mM EDTA, 1 mM CaCl_2_ for PPARδ-LBD/bezafibrate) at 4 or 20 °C for several weeks. In addition, PPARγ-LBD cocrystallization was performed in hanging-drop mixtures of 0.5 µL PPARγ-LBD (20 mg/mL in buffer A), 0.5 µL ligand (2 mM in buffer A), and 1 µL reservoir solution (0.1 M HEPES-NaOH [pH 7.5], 1.1 M trisodium citrate dihydrate for PPARγ-LBD/pemafibrate/SRC1; 0.1 M Tris [pH 8.0], and 1.1 M trisodium citrate dihydrate for PPARγ-LBD/bezafibrate/SRC1 or PPARγ-LBD/fenofibric acid/SRC1) at 20 °C for several weeks. The SRC1 peptide used for cocrystallization was LTERHKILHRLLQEG (amino acids [683–697] from GenScript). The obtained crystals were briefly soaked in a cryoprotection buffer (reservoir solution *+* 20% glycerol for PPARδ-LBD crystals and 30% glycerol for PPARγ-LBD crystals); afterward, these were flash cooled in a stream of liquid nitrogen until X-ray crystallography was conducted.

### 4.6. X-ray Diffraction: Data Collection and Model Refinement

Datasets were collected by BL-5A or BL-17A beamline at the Photon Factory (Ibaraki, Japan) using a synchrotron radiation of 1.0 Å. Diffraction data were collected at 0.1° oscillation per frame, and a total of 1800 frames (180°) were recorded for 1.0 Å X-ray crystallography. Data processing and scaling were carried out using XDS X-ray detector software and AIMLESS, respectively [6]. Resolution cutoff values (*R*_merge_ < 0.5, *R*_pim_ < 0.3, and completeness > 0.9) were set by the highest resolution shell. All structures were determined using molecular replacement in PHASER [48] with PDB ID: 2ZNQ for PPARδ-LBD/bezafibrate, 3GZ9 for PPARδ-LBD/pemafibrate, and 1WM0 for all PPARγ-LBD as the search model. Refinement was performed using iterative cycles of model adjustment in two programs: COOT and PHENIX [6]. The structures were constructed using PyMOL programs (http://www.pymol.org; accessed on 21 April 2020). All collection data and refinement statistics are summarized in Supplementary Table S1.

## Data Availability

Five novel PPARδ/γ-ligand structures reported in this study were deposited in PDB: 7WGL (PPARδ-LBD/bezafibrate), 7WGN (PPARδ-LBD/pemafibrate), 7WGO (PPARγ-LBD/bezafibrate/SRC1), 7WGP (PPARγ-LBD/fenofibric acid/SRC1), and 7WGQ (PPARγ-LBD/pemafibrate/SRC1).

## Supplementary Materials

The following supporting information can be downloaded at: https://www.mdpi.com/article/10.3390/ijms23094726/s1.
