# Supplementary material for: Functional and Structural Insights into Human PPARα/δ/γ Subtype Selectivity of Bezafibrate, Fenofibric Acid, and Pemafibrate
Source: Int J Mol Sci. 2022 Apr 25;23(9):4726. doi: 10.3390/ijms23094726 (PMC9102038; doi:10.3390/ijms23094726)
Supplement: Supplementary file 1 [file ijms-23-04726-s001.zip › ijms-1693348-supplementary.pdf]

Supplementary Materials

Functional and Structural Insights into Human PPARα/δ/γ Subtype Selectivity of Bezafibrate, Fenofibric Acid, and Pemafibrate

Akihiro Honda[1], Shotaro Kamata[1], Makoto Akahane[1], Yui Machida[1], Kie Uchii[1], Yui Shiiyama[1], Yuki Habu[1], Saeka Miyawaki[1], Chihiro Kaneko[1], Takuji Oyama[2], and Isao Ishii[1,]*

[1] *Department of Health Chemistry, Showa Pharmaceutical University, Machida, Tokyo 194-8543, Japan*

[2] *Faculty of Life and Environmental Sciences, University of Yamanashi, Kofu, Yamanashi 400-8510, Japan*

Contents

Supplementary Table S1. Data collection and refinement statistics (molecular replacement)

Supplementary Figure S1. Hydrophobic and hydrophilic surfaces of PPARα/δ/γ-LBD and the three fibrates with van der Waals spheres in cocrystal structures.

Supplementary Table S1

Data collection and refinement statistics (molecular replacement) (continues)

Protein	hPPARγ-LBD	hPPARγ-LBD	hPPARγ-LBD
Coactivator	SRC1	SRC1	SRC1
Ligand	Bezafibrate	Fenofibric acid	Pemafibrate
PDB ID	7WGO	7WGP	7WGQ
**Data collection**			
Space group	$P2_12_12_1$	$P2_12_12_1$	$P2_12_12_1$
Cell dimensions			
a, *b*, *c* (Å)	49.66, 64.95, 124.32	50.16, 64.46, 124.17	49.42, 63.32, 123.52
α, β, γ (°)	90.00, 90.00, 90.00	90.00, 90.00, 90.00	90.00, 90.00, 90.00
Resolution (Å)	46.12–2.36 (2.45–2.36)	46.51–2.53 (2.64–2.53)	45.89–2.43 (2.52–2.43)
R_{merge}	0.036 (0.332)	0.084 (0.374)	0.034 (0.339)
R_{pim}	0.016 (0.143)	0.036 (0.156)	0.014 (0.142)
$CC_{1/2}$	1.000 (0.984)	0.998 (0.984)	1.000 (0.977)
$I / \sigma I$	28.7 (4.9)	28.7 (4.9)	30.4 (5.1)
Completeness (%)	100.0 (99.9)	99.3 (95.4)	98.7 (98.0)
Redundancy	6.5 (6.3)	6.3 (6.5)	6.6 (6.7)
**Refinement**			
Resolution (Å)	39.449–2.360	44.718–2.530	38.587–2.430
No. reflections	17,145	13,870	14,938
R_{work} / R_{free}	0.1949/0.2253	0.2089/0.2686	0.2067/0.2518
No. atoms			
Protein	2,324	2,256	2,311
Ligand	25	66	36
Water	8	0	3
B-factors	58.93	64.81	60.45
Protein	58.76	64.61	60.37
Ligand	78.61	71.50	66.44
Water	46.23		47.32
Ramachandran plot (%)			
Favored	92.25	94.49	92.23
Allowed	5.28	3.68	4.95
Outliers	2.46	1.84	2.83
R.m.s. deviations			
Bond lengths (Å)	0.007	0.005	0.002
Bond angles (°)	1.01	0.74	0.51

Values in parentheses are for highest-resolution shell.

Supplementary Table S1

Data collection and refinement statistics (molecular replacement) (ends)

Protein	hPPARδ-LBD	hPPARδ-LBD
Coactivator	none	none
Ligand	Bezafibrate	Pemafibrate
PDB ID	7WGL	7WGN
**Data collection**		
Space group	$P2_1$	$P2\,2_12_1$
Cell dimensions		
a, *b*, *c* (Å)	39.48, 93.81, 96.23	37.77, 92.66, 96.76
α, β, γ (°)	90.00, 97.35, 90.00	90.00, 90.00, 90.00
Resolution (Å)	47.72–2.09 (2.15–2.09)	48.38–1.81 (1.85–1.81)
R_{merge}	0.036 (0.361)	0.032 (0.389)
R_{pim}	0.023 (0.231)	0.014 (0.163)
$CC_{1/2}$	0.999 (0.915)	1.000 (0.973)
$I / \sigma I$	16.2 (3.0)	29.0 (4.4)
Completeness (%)	98.8 (97.6)	99.8 (97.4)
Redundancy	3.5 (3.3)	6.6 (6.6)
**Refinement**		
Resolution (Å)	37.972–2.091	37.772–1.813
No. reflections	40,615	31,538
R_{work} / R_{free}	0.1944/0.2258	0.2261/0.2548
No. atoms		
Protein	4,156	2,126
Ligand	90	56
Water	48	58
B-factors	49.24	34.37
Protein	49.19	34.36
Ligand	54.65	35.82
Water	43.58	33.48
Ramachandran plot (%)		
Favored	97.41	96.47
Allowed	2.59	2.75
Outliers	0.00	0.78
R.m.s. deviations		
Bond lengths (Å)	0.009	0.002
Bond angles (°)	0.94	0.49

Values in parentheses are for highest-resolution shell.

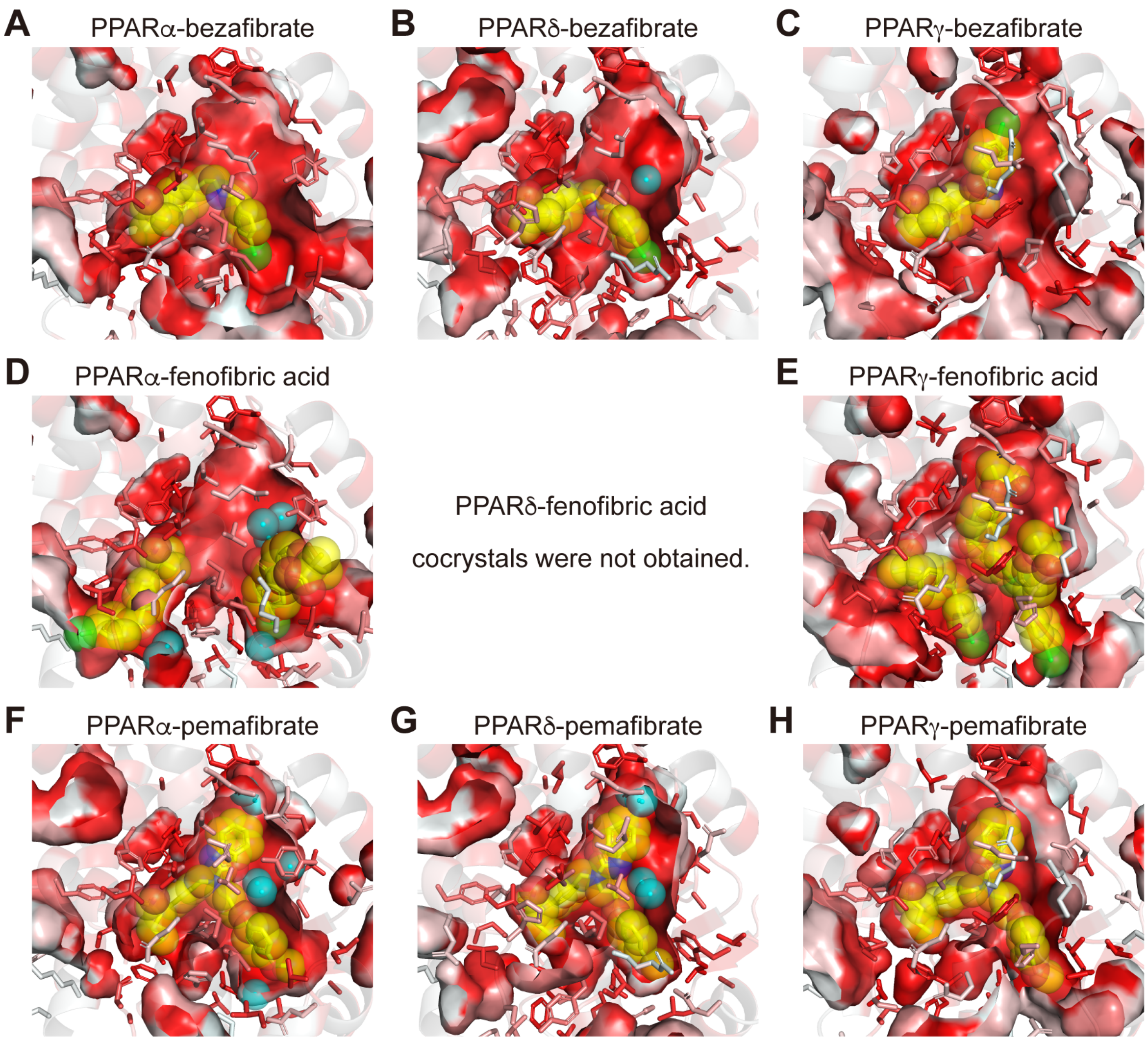

Supplementary Figure S1. Hydrophobic and hydrophilic surfaces of PPARα/δ/γ-LBD and the three fibrates with van der Waals spheres in cocrystal structures. Hydrophobic (*red*) and hydrophilic (*white*) surfaces of PPARα-LBD (A, D, and F), PPARδ-LBD (B and G), and PPARγ-LBD (C, E, and H) and bezafibrate (A–C), fenofibric acid (D and E), and pemafibrate (F–H) in van der Waals spheres (*yellow*) are illustrated. Water molecules and chlorides are shown in *cyan* and *green* spheres, respectively.
